# Epidural, Inadvertent Subdural, and Combined Epidural–Subdural Anesthesia in Lumbar Spine Surgery: A Retrospective Analysis

**DOI:** 10.3390/jpm14050486

**Published:** 2024-05-02

**Authors:** Seung Youn Kang, Hae Sun Cho, Jihwan Yi, Sung Chan Jung, Hyeun Sung Kim, Il Tae Jang, Hyun Kang

**Affiliations:** 1 Department of Anesthesiology and Pain Medicine, Nanoori Hospital Gangnam, Seoul 06048, Republic of Korea; 54kkang@naver.com (S.Y.K.); kreuz99@naver.com (H.S.C.); jihwanleemd@gmail.com (J.Y.); sc_chung@naver.com (S.C.J.); 2 Department of Neurosurgery, Cheongdam Harrison Hospital Gangnam, Seoul 06084, Republic of Korea; neurospinekim@gmail.com; 3 Department of Neurosurgery, Nanoori Hospital Gangnam, Seoul 06048, Republic of Korea; nanoori_research@naver.com; 4 Department of Anesthesiology and Pain Medicine, Chungang University College of Medicine, Seoul 06974, Republic of Korea

**Keywords:** anesthesia, epidural, deep sedation, fluoroscopy, subdural space

## Abstract

This study aimed to categorize contrast media images associated with epidural, subdural, and combined epidural–subdural anesthesia in patients who had undergone fluoroscopy-guided epidural anesthesia using contrast media combined with monitored anesthesia care (MAC) targeted at deep sedation, incorporating capnography over 5 years. Additionally, a correlation was established between the anesthetic effects and radiographic findings according to the categorized imaging appearances. This study included 628 patients who underwent endoscopic, open, or fusion surgery under epidural anesthesia at Nanoori Hospital in Gangnam between March 2018 and September 2023. Fluoroscopy-guided epidural anesthesia using contrast media combined with MAC and capnography was used. The dataset included detailed radiographic imaging, nursing, and anesthesia records. Distinct patterns of anesthesia administration were observed, with 49%, 19.6%, and 31% of patients receiving epidural, subdural, and combined epidural–subdural anesthesia, respectively. The incidence and duration of motor block were significantly different among the three groups. Additionally, subdural anesthesia displayed a higher incidence of motor block and a prolonged motor deficit duration than epidural anesthesia. Fluoroscopic guidance using a contrast medium for epidural and subdural anesthesia ensures precise space identification and prevents serious anesthetic complications. Our findings suggest the potential to achieve stable anesthesia, particularly using subdural and combined epidural–subdural anesthesia.

## 1. Introduction

Advancements and refinements in minimally invasive spinal surgery have tended toward either endoscopic or open minimally invasive procedures [1]. Feasible and effective anesthetic methods suitable for middle-aged individuals undergoing primary spine surgery and older patients undergoing revision procedures are urgently needed. Our previous research suggested that fluoroscopy-guided epidural anesthesia using contrast media, coupled with monitoring of deep sedation via a capnogram, is safe and viable, showing superiority in postoperative pain management and enhanced recovery [2,3].

Spinal anesthetic spaces can be classified into epidural, subdural, and subarachnoid spaces depending on the meningeal layer surrounding the spinal nerves [4]. Events, such as a midline gap in the ligamentum flavum or a deficiency resulting from prior spinal surgeries, can cause unclear tactile feedback when a needle is inserted through the ligamentum flavum [5]. In such situations, it is possible to discern the correct sensation only upon breaching the dura mater. Accurate needle tip placement is essential to achieve optimal therapeutic outcomes and prevent potential risks, including total spinal anesthesia or spinal infarction.

The complexities of the epidural space [6] are frequently overlooked and can manifest unpredictably in different ways during epidural anesthesia. Moreover, these complexities are affected by anatomic variances, the type and extent of pathology, the injectate flow, and the anesthesiologist’s proficiency [7]. Physicians must skillfully differentiate between the contrast flow patterns within the epidural, subdural, and subarachnoid spaces to identify potential dural punctures [8].

Recent research has challenged the concept of a natural subdural space, suggesting that what is conventionally identified as the subdural space may be an artifact resulting from dural–arachnoid separation due to needle injury or other mechanical causes [9]. This perspective introduces variability into the interpretation of subdural imaging patterns, prompting a shift from a belief in their natural existence to recognizing them as a potential procedural artifact [10,11]. Universally accepted diagnostic criteria for subdural injection are essential, as the imaging patterns previously assumed to represent the subdural space can vary. These patterns may range from “railroad track” images, such as elongated, thin, wispy columns observed within the dural sleeve in the anteroposterior view based on the prior assumption of the space’s existence [12], to a more defined, sausage-like mass of contrast visible in the anteroposterior view and an apparent ventral bulge in the lateral view, reflecting the evolving understanding of its nature [12,13].

We hypothesize that by comparing contrast media images associated with epidural, subdural, and combined epidural–subdural anesthesia, we can uncover and address specific relations between the anesthetic effects and radiographic findings. Thus, this study aimed to classify contrast media images associated with epidural, subdural, and combined epidural–subdural anesthesia in patients who had undergone fluoroscopy-guided epidural anesthesia using contrast media combined with monitored anesthesia care (MAC) targeted at deep sedation incorporating capnography over a period of 5 years. Additionally, the study aimed to establish a correlation between the anesthetic effects and radiographic findings according to the categorized imaging appearances.

## 2. Materials and Methods

This study was approved by the Institutional Review Board of Nanoori Hospital Medical Research Center (NR-IRB 2023-005). Given the retrospective nature of this study, requirement of the informed consent of the participants was waived. The research was conducted in accordance with the Ethical Principles for Medical Research Involving Human Subjects outlined in the 1975 Declaration of Helsinki (revised in 2013) and reported following the Strengthening of Reporting of Observational Studies in Epidemiology (STROBE) statement [14].

This study enrolled patients who underwent endoscopic, open, or fusion surgery under epidural anesthesia at the Nanoori Hospital in Gangnam between March 2018 and September 2023. The exclusion criteria were as follows: (1) patients whose anesthesia was switched from epidural to general due to unstable respiration and vital signs caused by high subarachnoid or combined subdural–subarachnoid anesthesia; (2) those with ambiguous, missing, or challenging-to-validate anesthesia, nursing, and radiographic image records. In cases where the interpretation of fluoroscopic images was unclear and ambiguous, it was not included in the data. Instead, a double-blind check was conducted, with two anesthesiologists reviewing the stored fluoroscopic images, and only those images that were agreed upon were included in the data.

### 2.1. Anesthetic Technique

Upon arrival in the operating room, the patients were placed in the prone position on a Wilson frame, blood pressure and oxygen saturation measurements were taken, and electrocardiography were performed. Their faces were placed in a hollowed-out rectangular face protection cushion. All the patients received a 3 L oxygen supply administered via a nasal cannula. After injecting the local anesthetic into the skin, under fluoroscopic guidance, a 20-gauge Tuohy needle was introduced into the nearest interlaminar epidural space cranial or caudal to the operative level, where the ligament flavum appears thick and taut in preoperative MRI. After identifying the epidural space using the loss-of-resistance technique and confirming the epidural placement using anteroposterior (AP) and lateral fluoroscopy with contrast media, a single injection of a concoction of 5–10 mL of 0.75% Ropivacaine with 1:200,000 epinephrine diluted in 5–10 mL of a radiocontrast dye (BONOREX^®^) (from 18 March 2018 to 23 May 2022) or 7.5–15 mL of 0.75% Ropivacaine with 1:200,000 epinephrine diluted in 2.5–5 mL of a radiocontrast dye (BONOREX^®^) (from 24 May 2022 to September 2023) was administered. Under fluoroscopic guidance, we infused the epidural canal until it reached the lower to mid-thoracic epidural space, based on the specific surgical level, to ensure adequate coverage of both the surgical field and the incisional dermatome. Fluoroscopy-guided neuraxial blockage (especially epidural anesthesia) for operation is a standard procedure in our country.

In cases of revision surgery with significant epidural adhesions inhibiting the spread of the dye cranially, we repeated this puncture superior to the level of epidural dye spread inhibition. Similarly, if the epidural canal did not fill caudally to the operative level because of severe stenosis, a second epidural injection was administered inferior to the operative level to ensure adequate epidural dye throughout the lumbar spine. If the anesthetic spread was too high, the patient was placed in the head-up position for at least 10 min at a 35° angle. Radiologic technicians obtained radiographs immediately and 10 min after epidural anesthesia using fluoroscopy in both the anteroposterior (AP) and lateral views.

**Figure 1:**
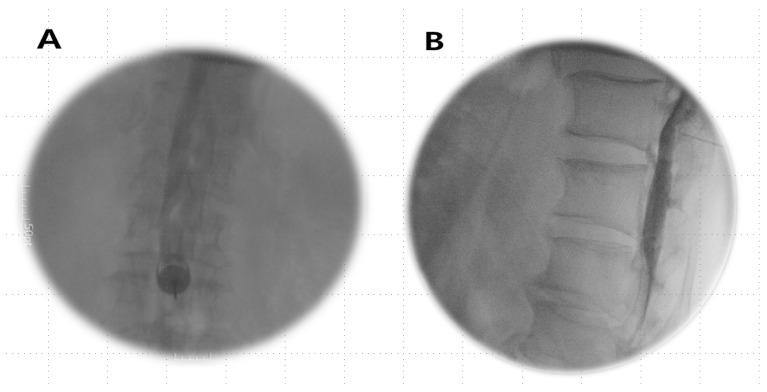
Subdural image. (**A**) AP view: a narrow column of contrast medium is confined to the central portion of the spinal canal and does not extend, outlining the exiting spinal nerve roots laterally. (**B**) Lateral view: the absence of CSF dilution and drainage; a longer duration persists, often remaining confined to the dorsal canal with a flat dorsal margin (dura mater) and an anterior bulging contrast (arachnoid mater) due to a smaller potential ventral subdural space.

**Figure 2:**
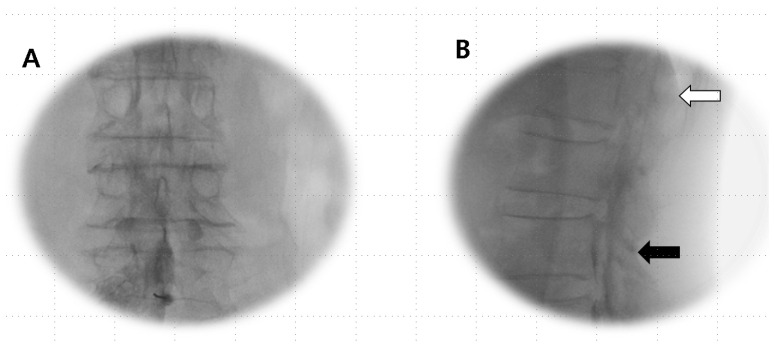
Combined epidural–subdural image. (**A**) AP view: imaging shows a thick, sausage-like mass of subdural contrast that transitions into a less dense epidural radiopaque image along the exiting nerve root, with the flow observed moving cranially. (**B**) Lateral view: the imaging reveals a progression from two thickened anterior–posterior columns positioned medially in the lower region (black arrow), transitioning to thinner, more peripheral anterior–posterior tracts (open arrow) that become more apparent in the epidural image as they flow cranially.

Anesthetics were administered if the Tuohy needle tip was situated within the epidural or subdural space (Figure 1A,B) or straddled between both spaces (Figure 2A,B).

**Figure 3:**
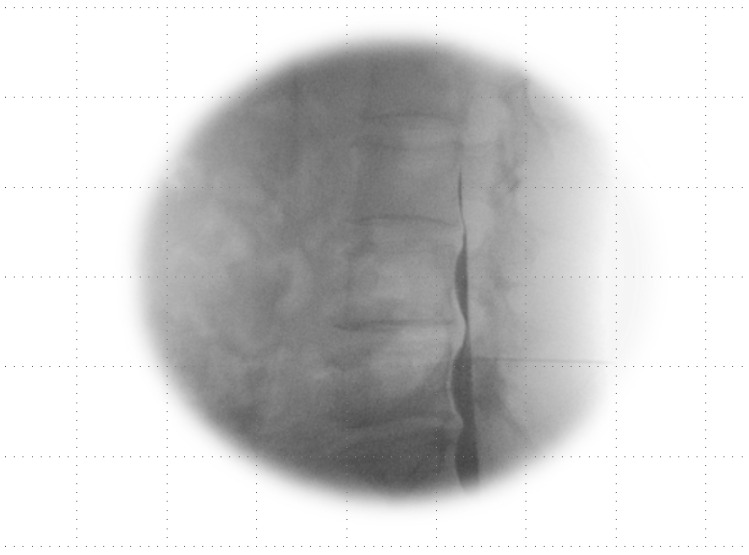
Subarachnoid image in lateral view: a single-layer image shows a pattern of hyperbaric contrast medium in the most ventrally dependent region featuring ventral undulations attributable to the arachnoid mater when in the prone position. There is a line of lucency between the contrast spread and the posterior vertebral body, suggestive of the area pertaining to the anterior epidural–subdural space.

**Figure 4:**
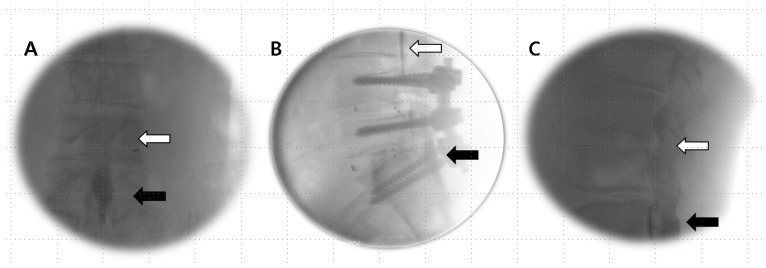
Combined subdural–subarachnoid image. (**A**) AP view: excessive injection into a thick, radiopaque subdural space (black arrow) ruptures the arachnoid mater, as evidenced by the cranial flow of CSF dilution (open arrow) characterized by a fleeting dispersal of the contrast medium. (**B**,**C**) Lateral view: the image shows a progression from two thickened posterior and/or anterior columns in the lower region (black arrow), transitioning to a single layer (open arrow) with a gradual change in the image pattern from local anesthetic in the posterior portion to hyperbaric contrast medium in the most ventrally dependent region, as they flow cranially.

If the Tuohy needle was positioned within the subarachnoid space (Figure 3) or straddled both the subdural and subarachnoid spaces (Figure 4A–C), it was removed, and epidural anesthesia was attempted at a different level.

After administering epidural anesthetics, we closely monitored the patients’ vital signs and comfort, especially their eyes and arms, for 5–10 min. If a patient was deemed stable and comfortable, a capnogram monitor was placed on their cheek or under their nose to monitor their respiratory status. The hollowed-out face cushion created a small pseudo-sealed chamber for the capnogram to obtain readings. We then initiated MAC targeted at deep sedation using a loading dose of 1 mcg/kg of dexmedetomidine (Precedex^®^, Pfizer, Pharmaceutical Korea, Seoul, Republic of Korea) for 10 min, followed by a maintenance dose of 0.6–0.7 mcg/kg/h of dexmedetomidine. Additionally, we infused 20 mcg/mL of remifentanil at a rate of 5–7 mL/h, along with 1–2 mg of midazolam based on the patient’s age, sex, and adjusted body weight. Hourly administration of an additional 1 mg dose of midazolam was continued. Further dosing of these medications was adjusted based on regular assessment of the patient’s respiratory status and degree of sedation.

When the patient became unresponsive to noxious stimuli, the oxygen level was increased to 6 L. A small pillow was placed below the rectangular face protection hollowed-out cushion to extend their neck to the sniffing position, thereby opening the airway. In the case of unstable breathing, as indicated by the capnogram, an oral or nasal airway was inserted, and oxygen was increased to 8–10 L using a mask to maintain stable saturation. During surgery, instances of hypotension (mean arterial pressure [MAP] < 75% of the baseline) were managed by administering fluid boluses. If necessary, interventions such as ephedrine (4–8 mg) or phenylephrine (50 mcg) were employed. In cases where these measures were insufficient, especially in patients taking long-term ARB or ACE inhibitors, terlipressin (1 mg), a vasopressin analog, was administered. Hypertension (MAP > 125% of the baseline) was assessed using nicardipine (500 mcg) or diltiazem (3 mg). Bradycardia (heart rate, <40 beats/min) was treated with atropine (0.5 mg)

After surgery, the patients were moved to the Post-Anesthesia Care Unit (PACU), where their vital signs were assessed, neurological examinations were performed, and their pain levels were evaluated using numerical rating scores (NRS). If the patient did not fully regain consciousness after 15 min of observation, we gradually administered 1/2 to 1 ampule of intravenous flumazenil to counteract any remaining effects of the midazolam. Once awake and responsive to verbal commands with accurately assessable motor function, the patients were transferred to the hospital ward. In the ward, their NRS scores and vital signs were reevaluated and neurological assessments repeated regularly.

### 2.2. Data Collection

We retrospectively reviewed the radiographic images, medical records, and clinical data extracted from the hospital’s electronic medical record (EMR) system. The data included details such as sex, age, height, immediate post-anesthesia posture, American Society of Anesthesiologists (ASA) physical status, history of previous spine surgery, operation duration, anesthesia duration, preoperative pain score, operated spine levels, extent of stenosis pathology, and type of surgical procedure. Additionally, the collected information included details regarding the spine level and laterality of needle insertion, the number of attempts during epidural anesthesia, the volume of epidural anesthetic injection, the spread level per volume of injectate, the sensory dermatome, and the occurrence and duration of motor block.

We gathered data on “analgesic spreads (anesthetic distance)” from the anesthesia records and data on “radiographic spread (distance)” and “changes in radiographic spread (distance)” 10 min after injection in both the neutral and head-up positions from the stored fluoroscopic radiographic images.

Analgesic spread (anesthetic distance) was defined as the measurement of the intervertebral levels from the epidural needle entry site to the sensory dermatome using an alcohol swab. Radiographic spread (distance) was defined as the calculation of the number of intervertebral levels covered by the contrast-enhanced anesthetic in the cephalad direction from the injection site based on the stored radiographic images obtained using fluoroscopy. Changes in the radiographic spread (distance) 10 min after injection involved identifying alterations in the radiographic distance observed 10 min after the epidural injection compared to that observed immediately after the injection. Furthermore, using the stored fluoroscopic radiographic images, we assessed the imaging patterns related to the epidural, subdural, and combined epidural–subdural configurations; instances of adhesion (where the anesthetic did not progress to the next level or spread to both sides); and instances of crossing the midline. Additionally, we documented the spread of contrast-enhanced anesthetics to the right and left sides of the midline.

### 2.3. Statistical Analysis

The patients were divided into three groups (epidural, subdural, and epidural–subdural anesthesia) according to the saved fluoroscopic radiographic images. For continuous variables, the data distribution was first evaluated for normality using the Shapiro–Wilk test. Normally distributed data were compared using one-way ANOVA with Scheffe’s post hoc test and presented as the mean ± standard deviation. Abnormally distributed data were compared using the Kruskal–Wallis test followed by the Mann–Whitney U test with Bonferroni correction and presented as the median (P_25_–P_75_). Descriptive variables were subjected to χ^2^ analysis or Fisher’s exact test, as appropriate, followed by the Bonferroni correction method to adjust the P-value, considering the potential false positive rate incurred by multiple comparisons. All the statistical analyses were performed using SPSS version 29.0.1.0 (IBM), and statistical significance was set at *p* < 0.05.

## 3. Results

Of the 628 patients, 308 received epidural anesthesia, 123 received subdural anesthesia, and 197 received combined epidural–subdural anesthesia, accounting for approximately 49%, 19.6%, and 31.4% of the sample, respectively. Statistically significant differences were observed in sex, age, height, history of previous surgery, the duration of surgery, and the duration of anesthesia among the three groups. Notably, the subdural anesthesia group showed a higher proportion of females and individuals with a history of previous surgeries, as well as a greater average age, a longer operation duration, and a longer anesthesia duration but a shorter average height (Table 1).

No differences were observed in the volume of contrast agent used in the epidurally injected anesthetic among the groups. The distance measured from the radiographic images using fluoroscopy (radiographic spread) was smaller in the subdural anesthesia group than in the epidural and combined epidural–subdural anesthesia groups. An examination of the changes in the radiographic distance 10 min post-injection, based on the patient’s position, indicated that the contrast-enhanced anesthetic spread in the cephalad increased by more than two spinal levels in the epidural and combined epidural–subdural groups (with average spreads of 2.16 ± 1.63 and 2.35 ± 2.03, respectively) compared to the subdural group, which showed an average spread of 0.22 ± 0.67 in the neutral position. In the head-up position, the contrast spread moved in the cephalad by approximately one level in the epidural and combined epidural–subdural groups (1.15 ± 1.79 and 1.27 ± 1.72, respectively). The subdural group had limited post-10 min image records, which made it difficult to draw conclusions. Furthermore, based on empirical knowledge, the onset of subdural anesthesia is slow, making it challenging to determine anesthetic dermatome results using the alcohol swab method.

The subdural space is formed according to the inherent adhesion between the dura and arachnoid mater, which can be disrupted by needle injury or other mechanical causes. This disruption poses a challenge in detecting adhesions during subdural anesthesia because the resulting space often does not show significant enlargement. Consequently, statistical analyses concerning the presence of adhesions in cases of subdural anesthesia were excluded from this study. Within the scope of this study, adhesions were defined according to the absence of cephalad or caudal spread of the contrast medium on both sides, which would typically form a “Christmas tree” pattern. This pattern was not observed in 44.2% and 41.1% of the patients in the epidural anesthesia and epidural–subdural anesthesia groups, respectively. However, in the subdural group, 21.1% of the cases were administered anesthetics at two sites, owing to either the anesthetic not passing through the previous surgical site or a site with severe stenosis.

In all the groups, the spread of the anesthetic was bilateral in most of the patients, irrespective of whether the injection was administered on the right or left side. However, unilateral spread was noted in 34.9%, 16.5%, and 27.3% of the patients in the epidural, subdural, and combined epidural–subdural groups, respectively.

Adhesions were notably prevalent in patients with severe stenosis and those with a history of spinal surgery. In the severe stenosis group, the adhesion rates were 85.7% and 65% under epidural and combined epidural–subdural anesthesia, respectively. In anticipation of potential issues with insufficient anesthesia for surgery, epidural anesthesia was administered at two distinct sites affected by severe stenosis in 33.3%, 53.8%, and 45% of the patients in the epidural, subdural, and combined epidural–subdural anesthesia groups, respectively.

Patients with previous spinal surgery exhibited nearly universal adhesions (87.3% in epidural surgery and 90.7% in combined epidural–subdural surgery), except in cases involving posterior spine fusion or transforaminal endoscopic lumbar discectomy (TELD) without decompression. Similar to the severe stenosis group, epidural anesthesia was administered at two opposing adhesion sites in 21.1% of the epidural anesthesia group, 27.7% of the subdural anesthesia group, and 35.2% of the combined epidural–subdural anesthesia group, in anticipation of inadequate anesthesia due to adhesions hindering the passage of anesthetics as affected by previous spine surgery.

Comparing the preoperative and postoperative conditions, the incidence of postoperative motor block or deficit due to anesthesia was 18%, 61%, and 55% in the epidural, subdural, and combined epidural–subdural anesthesia groups, respectively. The duration of motor deficit was 5.6 h (median, 5 h), 10 h (median, 8 h), and 9.2 h (median, 8 h) in the epidural, subdural, and combined epidural–subdural groups, respectively. Motor block in epidural anesthesia occurs mainly when a large amount of anesthetic is distributed longitudinally in a confined area owing to adhesion.

Excluding the cases of subarachnoid or combined subdural–subarachnoid anesthesia, no instances of motor block were observed in the epidural, subdural, or combined epidural–subdural groups at the time of evaluation of the anesthetic dermatome approximately 10 min after anesthesia (Table 2).

There was no evidence of a correlation between radiographic spread, analgesic spread from the anesthesia records, patient height, and anesthetic volume (Table 3).

There was no evidence of differences among the groups in terms of adverse events (Table 4).

## 4. Discussion

**Figure 5:**
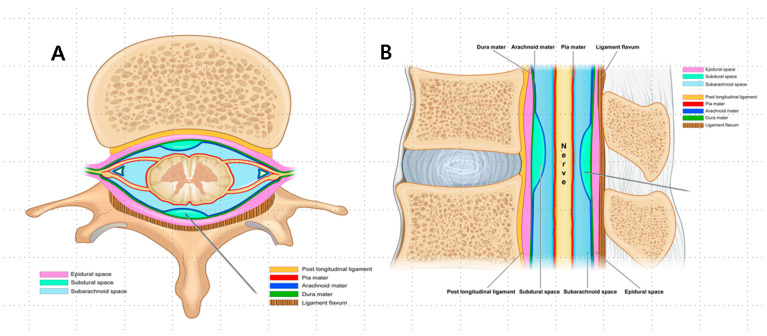
Epidural–inadvertent subdural–subarachnoid space (from outside to inside). (**A**) The coronal view: the subdural and subarachnoid spaces exist within the dural sleeve, while the epidural space exists beyond exiting roots. (**B**) The sagittal view: the epidural and subdural spaces are two distinct layers, whereas the subarachnoid space exhibits a single tubular morphology akin to a water tube.

This study, involving 628 patients, demonstrated varied patterns of anesthesia administration: a total of 49% (308 patients) received epidural anesthesia, 19.6% (123 patients) received subdural anesthesia, and 31% (197 patients) received a combination of epidural–subdural anesthesia. Despite the extensive experience of the anesthesiologists (>10 years), true epidural anesthesia was achieved in only approximately half of the cases. The observed incidence of subdural anesthesia of 19.6% exceeds the previously reported rates of 0.8–7% [15,16]. This may be influenced by the fact that 58.5% of the patients with subdural anesthesia had a history of spine surgery, which could affect the likelihood of the occurrence of subdural anesthesia. Additionally, the incidence of combined epidural–subdural anesthesia (31%) was higher than anticipated, which was attributed to the long, extended, and blunt tips of the Tuohy needle spanning both the epidural and subdural spaces. This phenomenon was explained by the possibility of the needle tip shifting during contrast-enhanced anesthetic injection or when entering the subdural space while handling the Tuohy needle bevel. In such cases, epidural–subdural anesthesia was dominant at lower levels near the needle entry site, transitioning to epidural-only anesthesia at higher levels as the anesthetic flowed cranially. These observations can be attributed to the anatomical features of the subdural and epidural spaces. The subdural space is practically absent, whereas the epidural space is anatomically present. Therefore, when administering anesthetics at comparable pressures, the flow of the anesthetic tends to favor the epidural space because of its actual presence, establishing a directional preference during the injection process. Spinal anatomy presents three potential anesthetic spaces—epidural, inadvertent subdural, and subarachnoid—because of the meninges surrounding the spinal nerves (Figure 5A,B). In 2002, Reina et al. reported that the subdural space, traditionally considered to exist, was not present, and that the dura–arachnoid interface was occupied by neuroglial cells [9]. However, the lower cohesive forces at this interface may result in mechanical separation between the dura and arachnoid matter, which are traditionally attached to each other, leading to fissure formation. Anesthetic agents entering these fissures gradually spread to areas with weaker bonds, creating a subdural space. Additionally, multiple secondary subdural spaces that are located closer to the dura mater and are more superficial than the primary subdural space can arise [9]. The terms “subdural” and “intradural” spaces have been used interchangeably in several studies, with some referring to secondary subdural spaces as intradural spaces [17]. However, the interchangeable use of the terms subdural space and intradural space might not be appropriate, as the spinal dura mater is a single layer, unlike the cranial dura mater, and the secondary subdural space is located at the dura–arachnoid interface and not between the dura layers [9].

**Figure 6:**
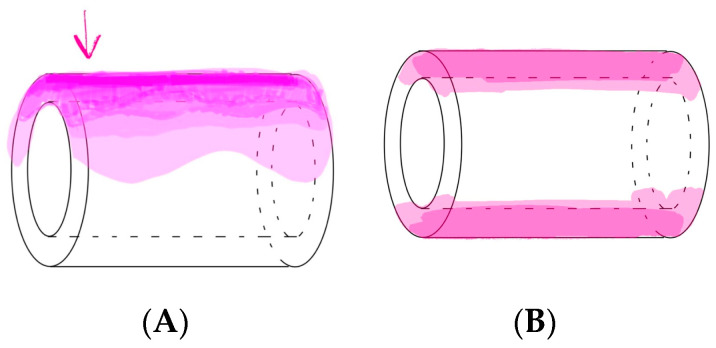
Changes in epidural images over time. (**A**) Immediate after epidural anesthesia: epidural pooling phase. (**B**) 10 min after epidural anesthesia: outermost linear, thin anterior, and/or posterior epidural images.

The anatomical characteristics of the subdural space give rise to varied imaging patterns, ranging from “railroad track” images to dense “sausage-like mass” contrasts. However, the lack of universally accepted diagnostic criteria for subdural injection stems from the nonexistent nature of the subdural space, which arises from iatrogenic fissures. The complexities of the epidural space go beyond initial recognition [6,18]. The contrast media patterns in epidural anesthesia exhibit variability owing to factors such as anatomical variations in the epidural space, the dynamics of the injectate flow, the expertise of anesthesiologists, and the nature and extent of spinal pathology [7]. Occasionally, epidural pooling and indentation during contrast-enhanced anesthesia may mimic a subdural pattern [12], particularly when a small volume of contrast-enhanced anesthetic is administered [6]. The most difficult aspect for us to distinguish was between epidural pooling, thick epidural spread, and subdural contrast images. Over time, epidural pooling contrast images undergo dilution and transform into outermost linear, thin anterior, and/or posterior epidural images (Figure 6A,B), whereas subdural contrast images remain unchanged over time, without dilution, and their shape remains constant. It was according to these time-dependent changes in the images that we were able to make some determinations. Additionally, based on our empirical knowledge, patients with epidural, subdural, or combined epidural–subdural imaging patterns report significant radiating leg pain upon injection. In contrast, those with combined subdural–subarachnoid or subarachnoid-only imaging patterns mainly complained of tingling sensations in their legs. Consequently, the patient symptoms during contrast-enhanced anesthesia injection and time-dependent changes in radiocontrast imaging proved valuable in identifying the injected space.

In our epidural group, we observed a lack of statistically significant correlation between the volume of contrast-enhanced anesthetic epidural injection, height, radiographic spread, and analgesic spread from the anesthesia records. This finding is consistent with prior research, which highlights the substantial variability in the anesthetized area following epidural block among patients [6,19]. Therefore, predicting anesthesia levels using height and volume for blind epidural techniques may be unreliable. Additionally, the traditional blind technique for epidural anesthesia, which relies on tactile cues, shows variable success, and the delayed onset of epidural anesthesia requires a 20–30 min confirmation period. Furthermore, epidural anesthesia involves a larger quantity of local anesthetic than spinal anesthesia, posing the risk of total spinal anesthesia if injected inadvertently into the subarachnoid space. However, fluoroscopy-guided epidural anesthesia using contrast media has emerged as a dependable and effective approach, offering the capability to predict the dermatomal distribution of anesthetic agents while being safe.

Our study suggests that for patients with severe stenosis or a history of spinal surgery, cranial application of epidural anesthesia at a specific site can prevent motor block and eliminate the need for a second anesthetic intervention due to adhesions. Likewise, administering anesthesia on the ipsilateral side of the pathology reduces the necessity for a second application due to adhesions.

The administration of anesthesia in the prone position may have influenced the outcomes in this retrospective study. A significant occurrence of motor block was noted in subdural anesthesia (61.5%), coupled with an extended duration of motor deficits (10 ± 5.31), which was 3.3-fold greater in size compared to that observed in epidural anesthesia (18.2%) and 1.8-fold longer in duration (5.6 ± 3.71). These results contradict those of previous research [20,21]. Even in the combined epidural–subdural group, where some of the anesthetic entered the subdural space, the incidence of motor block was 55.1%, and the duration of motor deficits was 9.2 ± 5.42, surpassing that observed in epidural anesthesia. The entrapment and retention of anesthetic within the equivocal subdural space, as opposed to the epidural or subarachnoid spaces, might conceivably impede its regular metabolism. The anatomical connection between the subdural space and cranial cavity creates a risk of total subdural anesthesia [22]. Additionally, the high incidence of motor blocks and prolonged deficit duration in subdural anesthesia up to the C3-5 level poses a potential threat to respiration. However, in this study, all the cases of subdural and combined epidural–subdural anesthesia being administered during surgery demonstrated no significant adverse effects, including on hemodynamic variables, when compared with epidural anesthesia. Therefore, this cautiously suggests that subdural anesthesia, despite being perceived as risky in spinal surgery, may demonstrate the potential to achieve stable anesthesia.

Our findings warrant careful interpretation considering several limitations. First, in our retrospective study, we did not conduct routine postoperative CT scans, which are essential to confirm the precise needle location and achieve accurate spatial localization [23]. Second, the lack of insurance coverage in the Korean medical system of central epidural anesthesia combined with MAC anesthesia, without BIS, SedLine, or entropy monitoring for sedation depth [24] or ANI or SPI for analgesic depth [25,26], raises concern about the overall safety and completeness of our approach. Third, MAC anesthesia in the prone position has the theoretical potential to cause respiratory depression, especially when using a continuous remifentanil infusion [27]. In our study, there was no dangerous apnea episode. The prone position might reduce upper airway obstruction risks compared to the supine position. However, anesthesiologists should take caution in that relying solely on capnogram monitoring and saturation may not ensure complete safety. Fourth, as with any retrospective study, our research may not fully encompass the complexities of various unmeasured variables, which require further elucidation of the results. Additionally, potentially confounding variables could have skewed the outcomes of our investigation. Fifth, there was a potential for selection bias due to the exclusion of ambiguous, missing, or difficult-to-validate records in the anesthesia, nursing, and radiographic images. This underscores the importance of a cautious interpretation of our findings. Sixth, because our data originated from a single institution, their direct applicability to other settings or populations may be limited. Therefore, a prospective large-scale multicenter study is imperative to validate these results and offer guidance for epidural anesthesia in patients undergoing endoscopic, open, or fusion surgery.

## 5. Conclusions

In this study, subdural and combined epidural–subdural anesthesia was observed in 51% (19.6% and 31.4%, respectively) of cases, mainly in patients with a history of spine surgery undergoing reoperation. Interestingly, subdural anesthesia showed a 3.3-fold higher incidence of postoperative motor block than traditional epidural anesthesia, with a prolonged duration exceeding the typical 4–5 h associated with subarachnoid anesthesia. Despite causing patient discomfort, subdural anesthesia demonstrated the potential to achieve stable anesthesia, contrary to the perceived risks of spine surgery. Further validation and clarification of these findings require larger prospective trials employing precise fluoroscopic views, accurate anesthetic records, and comprehensive postoperative recovery room and ward documentation.

## Figures and Tables

**Table 1:** Demographic characteristics.

		Type of Anesthesia			*p* Value
		Epidural (*n* = 308)	Subdural (*n* = 123)	Epidural–Subdural (*n* = 197)	
Sex	Male Female	173 (56)	46 (37)	97 (49)	0.002
		135 (44) †	77 (63) *	100 (51) *†	
Age (years)		58.23 ± 14.3 †	70.63 ± 11.2 *	64.84 ± 12.7 *†	<0.001
Height (cm)		163.9 ± 9 †	157.9 ± 10.1 *	161 ± 8.7 *†	<0.001
ASA physical status					0.213
I		3 (1)	-	3 (1.5)	
II		251 (81)	93 (76)	152 (77.6)	
III		54 (18)	29 (24)	41 (20.9)	
Previous spine surgery		71 (23) †	72 (59) *	54 (27) †	<0.001
Surgical procedure					
OPEN		156 (51)	55 (44.7)	97 (49.2)	0.315
Endo		125 (40.7)	54 (43.9)	73 (37.1)	0.315
OPEN fusion		25 (8)	12 (9.8)	26 (13.2)	0.315
Endo fusion		1 (0.3)	2 (1.6)	1 (0.5)	0.315
Operation time (min)		90.55 ± 40.9 †	105.41 ± 42.5 *	100.81 ± 46.6	0.002
Anesthesia time (min)		134.98 ± 43 †	150.24 ± 45.8 *	146.29 ± 48.6	0.002

* *p* < 0.05 compared with group E, † *p* < 0.05 compared with group S; OPEN: open discectomy from the 1 to 3 level and/or decompression; Endo: endoscopic discectomy from the 1 to 3 level and/or decompression; ASA, American Society of Anesthesiologists. Data are presented as numbers of patients (%) or mean ± SD.

**Table 2:** Anesthetic characteristics.

	Type of Anesthesia			*p* Value
	Epidural (*n* = 308)	Subdural (*n* = 123)	Epidural–Subdural (*n* = 197)	
Volume of epidural anesthetic injection	12.4 ± 3.53 (308)	12.6 ± 3.76 (97)	12.6 ± 3.9 (196)	0.852
Radiographic spread	6.1 ± 3.04 (150) †	4.6 ± 2.36 (66) *	6.5 ± 3.02 (111) †	<0.001
Analgesic spreads from anesthesia records	8.8 ± 2.25 (293) †	8 ± 2.7 (91) *	8.6 ± 2.31 (184)	0.013
Spread level per volume	0.73 (0.57–0.9)	0.7 (0.5–0.9)	0.72 (0.55–0.9)	
Changes in radiographic spread 10 min after injection				
Neutral	2.16 ± 1.63 (81), 2 (1–3) †	0.22 ± 0.67 (9), 0 (0–0) *	2.35 ± 2.03 (45), 2 (0.75–3.25) †	0.003
Head-up	1.15 ± 1.79 (48), 1 (0–2)	1 ± 1.73 (7), 0 (0–3)	1.27 ± 1.72 (48), 0.5 (0–2)	0.899
One-sided anesthesia (%)	34.9% (82) †	16.5% (15) *	27.3% (48) †	<0.001
Adhesion (%)	44.2% (130)	–	41.1% (81)	0.496
Anesthetic injection at two sites (%)	8.8% (27)	21.1% (26) *	17.8% (35) *	<0.001
Motor deficit (%)	18.2% (55)	61.5% (75) *	55.1% (108) *	<0.001
Motor deficit duration	5 (4–6) †	8 (6–14) *	8 (6–12) *	<0.001
Severe stenosis (%)	6.8% (21)	10.5% (13)	10.2% (20)	0.293
Anesthetic injection at two sites among severe stenosis pts (%)	33.3% (7)	53.8% (7)	45% (9)	0.483
Adhesion among severe stenosis pts (%)	85.7% (18)	–	65% (13)	0.123
Previous spine surgery	71 (23) †	72 (59) *	54 (27) †	<0.001
Anesthetic injection at two sites among previous surgery pts (%)	21.1% (15)	27.7% (20)	35.2% (19)	0.235
Adhesion among previous surgery pts (%)	87.3% (62)	–	90.7% (49)	0.73

Data are presented as means + SD, medians (P25–P75), or numbers of patients (%); * *p* < 0.05 compared with group E, † *p* < 0.05 compared with group S.

**Table 3:** Correlation of radiographic spread (distance), distance from the anesthesia record, patient height, and anesthetic volume.

	Anesthetic Volume	Height	Radiographic Spread	Analgesic Spreads
Anesthetic volume	1.000	−0.083	−0.041	0.028
		*p* = 0.163	*p* = 0.163	*p* = 0.163
Height		1.000	0.140	−0.133
			*p* = 0.163	*p* = 0.163
Radiographic spread (distance)			1.000	0.538
				*p* = 0.163
Analgesic spread (anesthetic distance)				1.000

Anesthetic volume: volume of contrast-enhanced anesthetic epidural injection; analgesic spread: analgesic spreads from anesthesia records.

**Table 4:** Hemodynamic Change.

	Type of Anesthesia			*p* Value
	Epidural (*n* = 308)	Subdural (*n* = 123)	Epidural-Subdural (*n* = 197)	
Hypotension	2 (1–2)	2 (1–2)	2 (1–2)	0.075
Vasopressor	0 (0–1)	0 (0–0)	0 (0–0)	0.125
Hypertension	0 (0–1)	0 (0–1)	1 (0–2)	0.132
Hypotensive agent	0 (0–1)	0 (0–1)	0 (0–1)	0.236

Data are presented as medians (P25–P75).

## Data Availability

The authors declare that the data in this research are available from the corresponding authors upon reasonable request.
